# Supplementary material for: Sexual harassment in secondary school: Prevalence and ambiguities. A mixed methods study in Scottish schools
Source: PLoS One. 2022 Feb 23;17(2):e0262248. doi: 10.1371/journal.pone.0262248 (PMC8865636; doi:10.1371/journal.pone.0262248)
Supplement: S1 File — (DOC) [file pone.0262248.s003.doc]

**S3 File – Parent and student study information and consent forms**

**Equally Safe At School evaluation**

**Parent information re student survey**

LOGOS – UNIVERSITY OF GLASGOW; MRC; CSO; ESAS

Dear Parent/Guardian,

A project called ‘Equally Safe At School’ (ESAS) is being piloted in this school during 2019-2020. ESAS focuses on gender equality and preventing gender-based violence (including physical, verbal and online sexual harassment and violence). You may be aware of reports of sexual harassment in many areas of life, including the film industry and other workplaces. Recent UK studies have found that sexual harassment is also common in schools.

The ESAS work is led by a co-ordinator from Rape Crisis, funded by the Scottish Government. It involves a range of school-based activities (a school self-assessment; policy reviews; an Action Group consisting of staff and students; staff training; student-led projects). The aim is to help young people become more aware, and less accepting of everyday sexism, and more able to challenge violence. ESAS also aims to increase staff confidence and skills in tackling inappropriate language and behaviours.

ESAS was piloted in 2 schools last year. We are University of Glasgow researchers studying this project to find out if it works and, if so, which of the school-based activities are most useful. We are also investigating whether it is realistic for schools to take on such activities and if it is possible for a school to continue with ESAS activities without support from an external co-ordinator.

To collect all the necessary information, we are asking all S2, S4 and S6 students to complete an online questionnaire and involving a small number of students in focus group discussions that will aim to better understand the situation in their school and their views on sexual harassment. All school staff will also be taking part in this research, via questionnaires and interviews.

We have received permission to conduct this research from the University of Glasgow, as well as <INSERT NAME> Council Education department and <INSERT NAME>, Head Teacher.

**This letter is to ask your permission for your child to take part in the student survey (further details overleaf).**

**Thank you for taking the time to read it.**

If you would like more information about this research, please contact:

Kirstin Mitchell or Helen Sweeting (Investigators),

MRC/CSO Social and Public Health Sciences Unit, University of Glasgow, 200 Renfield Street, Glasgow G2 3AX

Email: [kirstin.mitchell@glasgow.ac.uk](mailto:kirstin.mitchell@glasgow.ac.uk); helen.sweeting@glasgow.ac.uk

0141 353-7500

If you would like more information about ESAS, please contact:

<NAMES OF RELEVANT RAPE CRISIS SCOTLAND PERSONNEL>

Phone: Rape Crisis Scotland, Tara House, 46 Bath Street, Glasgow, G2 1HG

If you would like to speak to someone else not involved with this research or pursue any complaint, please contact:

<NAME OF COLLEGE OF SOCIAL SCIENCES ETHICS OFFICER>

University of Glasgow, St Andrew's Building, 11 Eldon Street, Glasgow, G3 6NH

Email: [XXX@glasgow.ac.uk](mailto:XXX@glasgow.ac.uk)

Phone: 0141-330-4699

| **Equally Safe At School Evaluation – student survey** |
| --- |
| **About the survey**  The survey will take place at school. It will be an online questionnaire, using mobile phones. We will provide the wifi connection via our own routers, so there will be no data charges. We will also provide tablets for pupils who do not have (functional) smartphones with them on the day.  The questionnaire will include questions on: experiences of ‘real world’ and online sexual harassment; gender equality and gender stereotypes; school, including whether school policies and the way it handles harassment are fair; some standard questions on health and mental well-being; awareness of ESAS; and some basic details such as whether they are male/female and their school year group. Note that we will not be asking for your child’s name and no information will be traceable back to individuals. It will take between 20 to 30 minutes to complete. Does my child have to take part? No. It is up to you and your child to decide. If, after reading this information letter, you feel that that you would prefer your child does not complete the questionnaire, please let us know by signing the attached ‘opt-out’ form, and asking your child to return it to us via their class teacher.  **If you are happy for your child to take part in the research, you don’t need to do anything**.  Before starting the questionnaire, we will ask your child to tick a box to show they understand what taking part involves, and that they are happy to do so. What if my child wants to withdraw from the study? Your child doesn’t have to answer any questions they don’t want to, and is free to withdraw at any time without having to explain their reasons. Even if they start the questionnaire, they are still free to change their mind.  **What will happen to the information my child gives?**  All data will be handled with the strictest confidence and in accordance with legal and ethical requirements for data storage. All data we collect is stored in securely locked filing cabinets and in password-protected databases. After the project is complete, all data will be securely archived and will be destroyed after ten years. Anonymised data will be available to other academic researchers designing similar projects, if we agree to their request. All our procedures comply with General Data Protection Guidelines.  **What are the benefits and risks of taking part?**  We have found that young people often enjoy taking part in research projects and focused discussions with their peers. It is an opportunity to use their experiences and views to contribute to a project that will benefit other young people in UK. There are no risks to taking part. Some of the questions ask about experiences of sexual harassment at school which young people may find uncomfortable. Before completing the questionnaire they will be reminded that they don’t have to answer any of the questions if they don’t want to. We need to ask these questions to understand the scale of the problem and whether things change as a result of the Equally Safe project.  The information will be used to develop and improve the ESAS project and in published journal articles and/or reports, including feedback to the school. |

**ESAS Student Questionnaire**

**Opt-Out Form - Parents/Guardians**

Please only return this form if you **DO NOT** want your child to take part in the student questionnaire.

(initial)

| 1. I have read and understood the information letter about the ESAS student questionnaire and know that I can get in touch with the researchers if I have more questions. |  |
| --- | --- |
| 1. I understand what the questionnaire is about, and why it is being carried out. |  |
| 1. I **DO NOT** give my permission for my child to complete a questionnaire. |  |

*You do not need to give a reason for withdrawing your child from the questionnaires but if you wish to give a reason please write below.*

|  |
| --- |

Your name (please print) Your child’s name (please print)

____________________________ ______________________________

Your signature Today’s date

____________________________ __________

Please give this slip to your child to return to their teacher if you **do not** want your child to take part.

**Equally Safe At School evaluation**

**Student information re student survey**

**LOGOS – UNIVERSITY OF GLASGOW; MRC; CSO; ESAS**

Dear Student,

A project called ‘Equally Safe At School’ (ESAS) is being run in this school during 2019-2020. ESAS focuses on promoting equality between boys and girls (‘gender equality’) and stopping everyday sexism.

Several activities are happening in the school as part of ESAS, including a student-staff Action Group and student projects.

We are University of Glasgow researchers studying ESAS to find out what students and staff think about it, whether it works and, if so, which of the activities are most useful.

We are asking all S2, S4 and S6 students to complete an online questionnaire and talking to small numbers of students about the questionnaire and their experiences of ESAS. School staff will also be helping us with this research.

We have received permission for this research from the University of Glasgow as well as <INSERT NAME> Council Education department and <INSERT NAME>, Head Teacher.

**This letter is to give you more information on the student survey (turn over for more information).**

**Thanks for reading.**

If you would like more information about this research, please contact:

Kirstin Mitchell or Helen Sweeting (Investigators),

MRC/CSO Social and Public Health Sciences Unit, University of Glasgow, 200 Renfield Street, Glasgow G2 3AX

Email: [kirstin.mitchell@glasgow.ac.uk](mailto:kirstin.mitchell@glasgow.ac.uk); helen.sweeting@glasgow.ac.uk

Telephone: 0141 353-7500

If you would like more information about the project ESAS, please contact:

<NAMES OF RELEVANT RAPE CRISIS SCOTLAND PERSONNEL>

Rape Crisis Scotland, Tara House, 46 Bath Street, Glasgow, G2 1HG

If you would like to speak to someone else not involved with this research or pursue any complaint, please contact:

<NAME OF COLLEGE OF SOCIAL SCIENCES ETHICS OFFICER>

University of Glasgow, St Andrew's Building, 11 Eldon Street, Glasgow, G3 6NH

Email: [XXX@glasgow.ac.uk](mailto:XXX@glasgow.ac.uk)

Telephone: 0141-330-4699

| **Equally Safe At School Evaluation – student survey** |
| --- |
| **About the survey**  The survey will take place at school. It will be an online questionnaire, using mobile phones. We will provide the wifi connection via our own routers, so there will be no data charges. We will also provide tablets for pupils who do not have (functional) smartphones with them on the day.  The questionnaire will include questions on: sexual harassment; gender equality and gender stereotypes; school and how it handles bullying and harassment; health and well-being; and some basic details such as whether you are male/female and your school year group. The questionnaire is anonymous, so we will not be asking for your name. It will take about 20-30 minutes to fill in.  **Do I have to complete the questionnaire?**  No. It is up to you to decide. Before starting the questionnaire, we will ask you to tick a box to show you understand what taking part involves, and that you are happy to do so. What if say yes but then change my mind? You don’t have to answer any questions you don’t want to and can change your mind about taking part at any time, even once you’ve started the questionnaire. If you choose not to take part, you don’t have to tell us why.  **What will happen to the information I give?**  We will look after the information very carefully. All the information we collect is stored in locked filing cabinets and in password-protected databases. The anonymous information will be stored safely for 10 years (these are University of Glasgow rules). It will be available to other academic researchers designing similar projects, but only with our permission. All our procedures comply with General Data Protection Guidelines. What’s in it for me? We have found that young people often enjoy taking part in research projects. It is an opportunity to use your experiences and views to contribute to a project that will help other young people in the UK.  The information will be used to develop and improve the ESAS project and in published articles and/or reports.  **Are there any risks in taking part?**  There are no risks in taking part. Some of the questions ask about experiences of sexual harassment at school which you might find uncomfortable. You don’t need to answer these questions if you don’t want to. We need to ask these questions to understand the scale of the problem and whether things change as a result of the Equally Safe project. |

**Equally Safe At School evaluation – Text at start of online student survey with consent box highlighted**


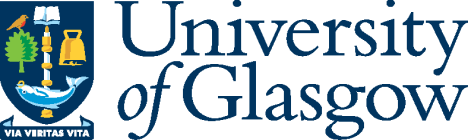

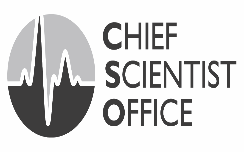

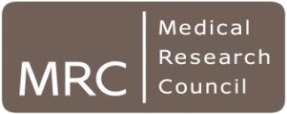


| A project called ‘Equally Safe At School’ (ESAS) is taking place in this school. It focuses on making your school a happier and safer place, where girls and boys can be treated with respect and in a fair way.  We are researchers from the University of Glasgow and we are studying the ESAS project. The survey asks questions about yourself, and how you feel in this school. And there are also questions about sexual harassment.  It is not a test. There are no right or wrong answers.  It is completely anonymous and confidential. We will not ask for your name during this survey, and therefore nobody will know what you have said. No one except researchers will see the answers.  And we will not share anything with anyone at school or at home.  We will store all the information you share with us in a safe place, and only use it for research. All the information will remain anonymous.  Please answer the questions as carefully and honestly as you can.  Remember that you don’t have to answer any questions you don’t want to, and you may stop at any time.  If you aren’t sure what a question means, please put your hand up and a researcher will come and help you.  **Please tick this box to show you have read the information above and are happy to fill in the questionnaire. *[box]*** |
| --- |

***NOTE – questionnaire on-line with paper copy as back-up***


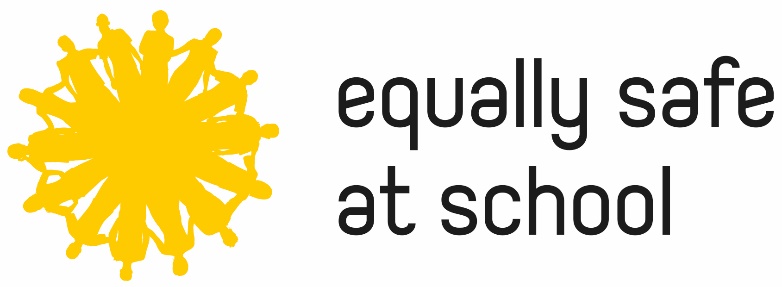


**Equally Safe At School evaluation**

**Parent information re student group interviews on ESAS experiences**

LOGOS – UNIVERSITY OF GLASGOW; MRC; CSO; ESAS

Dear Parent/Guardian,

A project called ‘Equally Safe At School’ (ESAS) is being piloted in this school during 2019-2020. ESAS focuses on gender equality and preventing gender-based violence (including physical, verbal and online sexual harassment and violence). You may be aware of reports of sexual harassment in many areas of life, including the film industry and other workplaces. Recent UK studies have found that sexual harassment is also common in schools.

The ESAS work is led by a co-ordinator from Rape Crisis, funded by the Scottish Government. It involves a range of school-based activities (a school self-assessment; policy reviews; an Action Group consisting of staff and students; staff training; student-led projects). The aim is to help young people become more aware, and less accepting of everyday sexism, and more able to challenge violence. ESAS also aims to increase staff confidence and skills in tackling inappropriate language and behaviours.

ESAS was piloted in 2 schools last year. We are University of Glasgow researchers studying this project to find out if it works and, if so, which of the school-based activities are most useful.

To collect all the necessary information, we are asking all students all S2, S4 and S6 classes to complete an online questionnaire and involving a small number of students in focus group discussions that will aim to better understand the situation in their school and their views on sexual harassment. All school staff will also be taking part in this research, via questionnaires and interviews.

We have received permission to conduct this research from the University of Glasgow, as well as <INSERT NAME> Council Education department and <INSERT NAME>, Head Teacher.

**This letter is to ask your permission for your child to take part a in group interview to talk about their experiences of the ESAS project in their school (further details overleaf).**

**Thank you for taking the time to read it.**

If you would like more information about this research, please contact:

Kirstin Mitchell or Helen Sweeting (Investigators),

MRC/CSO Social and Public Health Sciences Unit, University of Glasgow, 200 Renfield Street, Glasgow G2 3AX

Email: [kirstin.mitchell@glasgow.ac.uk](mailto:kirstin.mitchell@glasgow.ac.uk); helen.sweeting@glasgow.ac.uk

Phone: 0141 353-7500

If you would like more information about ESAS, please contact:

<NAMES OF RELEVANT RAPE CRISIS SCOTLAND PERSONNEL>

Phone: Rape Crisis Scotland, Tara House, 46 Bath Street, Glasgow, G2 1HG

If you would like to speak to someone else not involved with this research or pursue any complaint, please contact:

<NAME OF COLLEGE OF SOCIAL SCIENCES ETHICS OFFICER>

University of Glasgow, St Andrew's Building, 11 Eldon Street, Glasgow, G3 6NH

Email: [XXX@glasgow.ac.uk](mailto:XXX@glasgow.ac.uk)

Phone: 0141-330-4699

| **Equally Safe At School Evaluation – Group interviews about ESAS** |
| --- |
| **What is my child being asked to do?**  The team who designed ESAS want to know how well it worked and what students thought of the activities. We are keen to hear from a range of students, whether or not they had a big involvement in any of the activities. Their view will help the team improve the project in the future.  We would therefore like to invite your child to take part in group interviews to talk about the ESAS activities, or any changes they have noticed within the school – and to give us their ideas about how the project could be improved. Teachers will assist us in nominating students to take part. We are looking to interview a wide range of students (boys and girls; younger and older) with different experiences and views on the Equally Safe project. The group discussions will take about 45 minutes and would take place during school time. It would be led by Carolyn Blake, Kirstin Mitchell or Helen Sweeting, all of whom are experienced in doing research with young people. Does my child have to take part? No. It is up to you and your child to decide. If, after reading this information letter, you feel that that you would prefer your child does not take part in an interview, please let us know by signing the attached ‘opt-out’ form, and asking your child to return it to us via their class teacher.  **If you are happy for your child to take part in the research, you don’t need to do anything**.  Before starting the group interview, we will ask your child to sign a consent form to show they understand what taking part involves, and that they are happy to do so. What if my child wants to withdraw from the study? Your child doesn’t have to answer any questions they don’t want to, and is free to withdraw at any time without having to explain their reasons. Even if they start the group interview, they are still free to change their mind. What will happen to the information my child gives? We ask that everyone taking part in the group interviews is respectful towards the views of others and treats what is said confidentially. We will maintain confidentiality as far as possible, but if we hear anything which makes us worried that someone might be in danger of harm, we might have to inform relevant agencies of this.  Discussions will be audio recorded, with participants’ permission, transcribed for analysis, and all identifying details removed. Although we may use your child’s words in publications and reports, we will not use their real name, meaning these words cannot be traced back to an individual.  All data will be handled with the strictest confidence and in accordance with legal and ethical requirements for data storage. All data we collect is stored in securely locked filing cabinets and in password-protected databases. After the project is complete, all data will be securely archived and will be destroyed after ten years. Anonymised data will be available to other academic researchers designing similar projects, if we agree to their request. All our procedures comply with General Data Protection Guidelines.  **What are the benefits and risks of taking part?**  We have found that young people often enjoy taking part in research projects and focused discussions with their peers. It is an opportunity to use their experiences and views to contribute to a project that will benefit other young people in UK. There are no risks to taking part. Some of the discussion will cover sexual harassment at school which young people may find uncomfortable. The discussion will be about general issues and not personal experiences. We need to ask these questions to understand the scale of the problem and whether things change as a result of the Equally Safe project.  The information from this evaluation will be used to develop and improve the ESAS project and in published journal articles and/or reports, including feedback to the school. |

**ESAS Student Group Interviews**

**Opt-Out Form - Parents/Guardians**

Please only return this form if you **DO NOT** want your child to take part in the student interview.

(initial)

| 1. I have read and understood the information letter about the ESAS student group interviews and know that I can get in touch with the researchers if I have more questions. |  |
| --- | --- |
| 1. I understand what the interviews are about, and why they are being carried out. |  |
| 1. I **DO NOT** give my permission for my child to take part in an interview. |  |

*You do not need to give a reason for withdrawing your child from the questionnaires but if you wish to give a reason please write below.*

|  |
| --- |

Your name (please print) Your child’s name (please print)

____________________________ ______________________________

Your signature Today’s date

____________________________ __________

Please give this slip to your child to return to their teacher if you **do not** want your child to take part.

**Equally Safe At School evaluation**

**Student information re student focus groups on ESAS experiences**

**LOGOS – UNIVERSITY OF GLASGOW; MRC; CSO; ESAS**

Dear Student,

A project called ‘Equally Safe At School’ (ESAS) is being run in this school during 2019-2020. ESAS focuses on promoting equality between boys and girls (‘gender equality’) and stopping everyday sexism.

Several activities are happening in the school as part of ESAS, including a student-staff Action Group and student projects.

We are University of Glasgow researchers studying ESAS to find out what students and staff think about it, whether it works and, if so, which of the activities are most useful.

We are asking all students from S2, S4 and S6 classes to complete an online questionnaire and talking to small numbers of students about the questionnaire and their experiences of ESAS. School staff will also be helping us with this research as well.

We have received permission for this research from the University of Glasgow, and <INSERT NAME> Council Education department and <INSERT NAME>, Head Teacher.

**This letter is to give you more information about student group interviews to talk about your experiences of the ESAS project in your school (turn over for more information).**

**Thanks for reading.**

If you would like more information about this research, please contact:

Kirstin Mitchell or Helen Sweeting (Investigators),

MRC/CSO Social and Public Health Sciences Unit, University of Glasgow, 200 Renfield Street, Glasgow G2 3AX

Email: [kirstin.mitchell@glasgow.ac.uk](mailto:kirstin.mitchell@glasgow.ac.uk); helen.sweeting@glasgow.ac.uk

Phone: 0141 353-7500

If you would like more information about the project ESAS, please contact:

<NAMES OF RELEVANT RAPE CRISIS SCOTLAND PERSONNEL>

Rape Crisis Scotland, Tara House, 46 Bath Street, Glasgow, G2 1HG

If you would like to speak to someone else not involved with this research or pursue any complaint, please contact:

<NAME OF COLLEGE OF SOCIAL SCIENCES ETHICS OFFICER>

University of Glasgow, St Andrew's Building, 11 Eldon Street, Glasgow, G3 6NH

Email: [XXX@glasgow.ac.uk](mailto:XXX@glasgow.ac.uk)

Telephone: 0141-330-4699

| **Equally Safe At School Evaluation – student interviews** |
| --- |
| **What am I being asked to do?**  The team who designed ESAS want to know how well it worked and what students thought of the activities. We are keen to hear from a range of students, whether or not they had a big involvement in any of the activities. Their views will help the team improve the project in the future.  We would therefore like to invite you to join a small group of students to talk about the ESAS activities, or any changes you have noticed in the school – and to give us your ideas about how the project could be improved. The teachers have assisted us in nominating students to take part. We are looking to interview a wide range of students (boys and girls; younger and older) with different experiences and views on the Equally Safe project. The group discussions will take about 45 minutes and would take place during school time. The discussion would be led by a researcher from the University of Glasgow, either, Carolyn Blake, Kirstin Mitchell, Helen Sweeting. We have all done research with young people before.  **Do I have to take part in an interview?**  No. It is up to you to decide. Before starting the group interview, we will ask you sign a consent form to show you understand what taking part involves, and that you are happy to do so. What if say yes but then change my mind? You don’t have to answer any questions you don’t want to and can change your mind about taking part at any time, even once you’ve started the interview. If you choose not to take part, you don’t have to tell us why. What will happen to the information I give? We ask that everyone taking part in the session is respectful of the views of others and does not repeat any of the discussion to people outside of the group. We will keep what you say to us confidential (private), but if we hear anything which makes us worried that you or someone else might be in danger of harm, we might have to tell someone.  We will audio record the session, if you give us permission, and then type up what was said, making sure we remove all real names. Although we may use your words in written reports, we will not use your real name, so your words cannot be traced back to you.  We will look after the information very carefully. All the information we collect is stored in locked filing cabinets and in password-protected databases. The anonymous information will be stored safely for 10 years (these are University of Glasgow rules). It will be available to other academic researchers designing similar projects, but only with our permission. All our procedures comply with General Data Protection Guidelines. What’s in it for me? We have found that young people often enjoy taking part in research projects and these sorts of discussion. It is an opportunity to use your experiences and views to contribute to a project that will help other young people in the UK.  The information will be used to develop and improve the ESAS project and in published articles and/or reports.  **Are there any risks in taking part?**  There are no risks in taking part. Some of the discussion will cover sexual harassment at school which you may find uncomfortable. We will be discussing issues in general, and not personal experiences. We need to ask these questions to understand the scale of the problem and whether things change as a result of the Equally Safe project. |


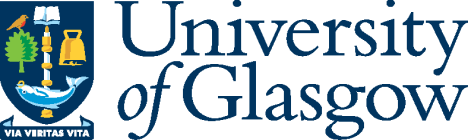

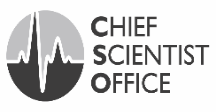

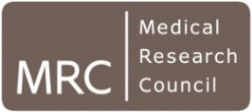


**Equally Safe At School evaluation**

**Student group interview consent form**

|  | **Please tick ONE BOX for each statement** | **YES** |  | **NO** |
| --- | --- | --- | --- | --- |
| **1** | I understand about the ESAS research from reading the information sheet, or talking to a researcher.  I have had the chance to think about taking part and to ask questions. |  |  |  |
|  |  |
|  |  |
|  |  |  |  |  |
| **2** | I understand that taking part is my choice.  I know that I can stop or withdraw at any time and that I don’t have to give a reason if I decide to stop. |  |  |  |
|  |  |  |  |  |
| **3** | I agree to a sound recording being made of what I say.  I understand that the information I give will be stored safely at the University of Glasgow. |  |  |  |
|  |  |
|  |  |
|  |  |  |  |  |
| **4** | I know that short quotes of what I say may be used in reports, research articles or talks.  I know that if any of these use my words, they will not include anything that could identify me. |  |  |  |
|  |  |  |
|  |  |  |
|  |  |  |  |  |
| **5** | I understand that my name will not appear in any reports, research articles or talks. |  |  |  |
|  |  |  |  |  |
| **6** | I know the university researchers have to follow strict rules about confidentiality.  I know the information I give will be available to other genuine researchers designing similar projects if the University of Glasgow researchers give their permission, and that it will be destroyed after 10 years. |  |  |  |
|  |  |
|  |  |

**I agree to take part in the study.**

______________________ ______________ ________________

Participant name Signature ……. Date

______________________ ______________ ________________

Researcher name Signature ……. Date
