# Supplementary material for: Sexual harassment in secondary school: Prevalence and ambiguities. A mixed methods study in Scottish schools
Source: PLoS One. 2022 Feb 23;17(2):e0262248. doi: 10.1371/journal.pone.0262248 (PMC8865636; doi:10.1371/journal.pone.0262248)
Supplement: S1 Table — (DOC) [file pone.0262248.s004.doc]

**S4 File - SUPPLEMENTARY TABLE 1: Sexual harassment items – self-reported victimization and perpetration - descriptive data**

|  |  | **VICTIMIZATION (past 3 months)** | | | | |  | **PEPRETRATION (past 3 months)** | | | | |
| --- | --- | --- | --- | --- | --- | --- | --- | --- | --- | --- | --- | --- |
| **Survey items** | **Item type #** | **(Valid N)** | **Often / Occasionally**  **(%)** | **Rarely**  **(%)** | **Never**  **(%)** | **Not sure**  **(%)** |  | **(Valid N)** | **Often / Occasionally**  **(%)** | **Rarely**  **(%)** | **Never**  **(%)** | **Not sure**  **(%)** |
| 1-Made sexual jokes, gestures or looks | V/V | (617) | 23.7 | 22.2 | 45.4 | 8.8 |  | (608) | 11.7 | 12.2 | 73.2 | 3.0 |
| 2-Showed you or sent you sexual images or messages that you did not want to see | V/V | (612) | 10.3 | 14.2 | 69.8 | 5.7 |  | (606) | 1.0 | 1.7 | 94.7 | 2.6 |
| 3-Wrote sexual messages / graffiti about you in public places (eg. on toilet walls, in changing rooms). | V/V | (613) | 2.8 | 4.7 | 85.6 | 6.9 |  | (607) | 1.3 | 0.8 | 95.9 | 2.0 |
| 4-Spread sexual rumours about you online or in person | V/V | (614) | 7.7 | 8.8 | 77.0 | 6.5 |  | (606) | 1.3 | 2.8 | 93.2 | 2.6 |
| 5-Said you were gay or lesbian, in a hurtful way | V/V | (613) | 11.7 | 9.6 | 73.6 | 5.1 |  | (603) | 4.6 | 3.5 | 88.9 | 3.0 |
| 6-Spied on you as you dressed or showered at school | PC/P | (610) | 2.3 | 1.0 | 88.5 | 8.2 |  | (603) | 0.0 | 0.0 | 98.0 | 2.0 |
| 7-Flashed /‘mooned’ at you (showed their private parts or exposed themselves) | V/V | (607) | 7.4 | 6.3 | 82.9 | 3.5 |  | (605) | 1.5 | 3.0 | 93.4 | 2.1 |
| 8-Touched, grabbed, or pinched you in a sexual way | PC/P | (612) | 6.9 | 10.3 | 79.7 | 3.1 |  | (602) | 1.7 | 2.2 | 93.9 | 2.3 |
| 9-Brushed up against you in a sexual way on purpose | PC/P | (609) | 6.6 | 5.7 | 81.4 | 6.2 |  | (605) | 1.0 | 1.0 | 95.7 | 2.3 |
| 10-Pulled at your clothing in a sexual way | PC/P | (610) | 2.6 | 4.8 | 89.3 | 3.3 |  | (603) | 0.5 | 1.2 | 96.5 | 1.8 |
| 11-Pulled off or down your clothing | PC/P | (613) | 2.3 | 2.4 | 92.5 | 2.8 |  | (603) | 0.2 | 1.5 | 96.5 | 1.8 |
| 12-Blocked your way or cornered you in a way that made you feel uncomfortable | PC/P | (610) | 3.4 | 9.2 | 84.1 | 3.3 |  | (603) | 0.0 | 0.8 | 96.7 | 2.5 |
| 13-Made you kiss him / her | PC/P | (613 | 2.4 | 3.3 | 91.4 | 2.9 |  | (601) | 0.2 | 0.5 | 97.2 | 2.2 |
| 14-Made you do something sexual other than kissing (like touching their private parts) | PC/P | (612) | 1.8 | 1.8 | 93.5 | 2.9 |  | (601) | 0.2 | 0.0 | 98.0 | 1.8 |
| 15-Taken a picture to see under your clothes, eg. up your skirt or down your shirt | PC/P | (612) | 0.8 | 1.0 | 94.1 | 4.1 |  | (602) | 0.3 | 0.0 | 98.0 | 1.7 |
| 16-Forwarded a naked or sexual picture of you to others, without your agreement | PC/P | (613) | 1.5 | 2.9 | 92.0 | 3.6 |  | (604) | 0.5 | 0.2 | 97.4 | 2.0 |
| 17-Pressured you to send them a naked (nude) or sexual picture of yourself | PC/P | (613) | 4.4 | 5.4 | 87.6 | 2.6 |  | (602) | 0.5 | 0.0 | 97.8 | 1.7 |

# V/V = visual or verbal; PC/P = physical contact or personally-invasive.
