# Supplementary material for: Sexual harassment in secondary school: Prevalence and ambiguities. A mixed methods study in Scottish schools
Source: PLoS One. 2022 Feb 23;17(2):e0262248. doi: 10.1371/journal.pone.0262248 (PMC8865636; doi:10.1371/journal.pone.0262248)
Supplement: S2 Table — (DOC) [file pone.0262248.s005.doc]

**S5 File - SUPPLEMENTARY TABLE 2: Composite variables: basic frequencies**

|  | **VICTIMIZATION** | |  | **PERPETRATION** | |
| --- | --- | --- | --- | --- | --- |
|  | **N** | ***(valid %)*** |  | **N** | ***(valid %)*** |
| **Any visual/verbal#** |  |  |  |  |  |
| Yes | 367 | *(64.7)* |  | 168 | *(29.0)* |
| No | 200 | *(35.3)* |  | 411 | *(71.0)* |
| Missing | 71 | *-* |  | 59 | *-* |
| **Any contact/personally-invasive#** |  |  |  |  |  |
| Yes | 193 | *(34.3)* |  | 36 | *(6.4)* |
| No | 369 | *(65.7)* |  | 530 | *(93.6)* |
| Missing | 76 |  |  | 72 | *-* |
| **Any of either type** |  |  |  |  |  |
| Yes | 383 | *(68.3)* |  | 169 | *(30.0)* |
| No | 178 | *(31.7)* |  | 395 | *(70.0)* |
| Missing | 77 | *-* |  | 74 | *-* |

# any visual/verbal = any of 6 items; any contact/personally-invasive = any of 11 items
