# Supplementary material for: Sexual harassment in secondary school: Prevalence and ambiguities. A mixed methods study in Scottish schools
Source: PLoS One. 2022 Feb 23;17(2):e0262248. doi: 10.1371/journal.pone.0262248 (PMC8865636; doi:10.1371/journal.pone.0262248)
Supplement: S3 Table — (DOC) [file pone.0262248.s006.doc]

**S6 File - SUPPLEMENTARY TABLE 3: Cross-tabulations of visual/verbal and contact/personally-invasive victimization and perpetration – numbers (and valid cell percentages) and Chi-square**

|  |  | **VICTIMIZATION** | | |  | **PERPETRATION** | | |
| --- | --- | --- | --- | --- | --- | --- | --- | --- |
|  |  | **Visual/verbal** | | |  | **Visual/verbal** | | |
|  |  | **Yes**  **N**  **(Total %)** | **No**  **N**  **(Total %)** | **Missing**  **N** |  | **Yes**  **N**  **(Total %)** | **No**  **N**  **(Total %)** | **Missing**  **N** |
| **Contact/personally-invasive** | **Yes** | 177  *(33.7%)* | 14  *(2.7%)* | 2 |  | 35  *(6.3%)* | 1  *(0.2%)* | 0 |
|  | **No** | 156  *(29.7%)* | 178  *(33.9%)* | 35 |  | 120  *(21.8%)* | 395  *(71.7%)* | 15 |
|  | **Missing** | 34 | 8 | 34 |  | 13 | 15 | 44 |
|  |  |  |  |  |  |  |  |  |
| ***Chi-sq (based on valid data) (p)*** |  | *110.7 (<0.001)* | |  |  | *90.9 (<0.001)* | |  |
|  |  |  |  |  |  |  |  |  |
