# Supplementary material for: Sexual harassment in secondary school: Prevalence and ambiguities. A mixed methods study in Scottish schools
Source: PLoS One. 2022 Feb 23;17(2):e0262248. doi: 10.1371/journal.pone.0262248 (PMC8865636; doi:10.1371/journal.pone.0262248)
Supplement: S4 Table — (DOC) [file pone.0262248.s007.doc]

**S7 File - SUPPLEMENTARY TABLE 4: Cross-tabulation of composite victimization and perpetration variables**

|  | **PERPETRATION** | | |
| --- | --- | --- | --- |
|  | **None** | **Visual/verbal only** | **Any contact/**  **personally-invasive** |
|  | **Row %** | **Row %** | **Row %** |
| **VICTIMIZATION** |  |  |  |
| None | 94.7 | 5.3 | 0.0 |
| Visual/verbal only | 67.6 | 31.0 | 1.4 |
| Any contact/personally-invasive | 50.9 | 29.6 | 19.5 |
| ***Chi-sq (p)*** | *108.6 (<0.001)* | | |
